# Identification of optimal surgical plan for treatment of extraocular muscle damage in thyroid eye disease patients based on computational biomechanics

**DOI:** 10.3389/fbioe.2022.969636

**Published:** 2023-01-10

**Authors:** Byeong Cheol Jeong, Chiseung Lee, Jungyul Park, Dongman Ryu

**Affiliations:** ^1^ Department of Biomedical Engineering, Graduate School, Pusan National University, Busan, South Korea; ^2^ Department of Convergence Medicine, School of Medicine, Pusan National University, Busan, South Korea; ^3^ Department of Biomedical Engineering, School of Medicine, Pusan National University, Busan, South Korea; ^4^ Biomedical Research Institute, Pusan National University Hospital, Busan, South Korea; ^5^ Department of Ophthalmology, School of Medicine, Pusan National University Hospital, Busan, South Korea; ^6^ Medical Research Institute, Pusan National University, Busan, South Korea

**Keywords:** thyroid eye disease, orbital decompression, hypertrophy, inferior rectus muscle, recurrence, finite element analysis

## Abstract

This study replicated the behavior of intraorbital tissue in patients with thyroid eye disease (TED) based on finite element analysis for general orbital decompression risk evaluation in thyroid eye disease patients. The orbit and intraorbital tissues of thyroid eye disease patients who underwent orbital decompression were modeled as finite element models. The stress was examined at specific locations of the removed orbital wall of a thyroid eye disease patient with undergone orbital decompression, and its variation was analyzed as a function of the shape and dimension (to be removed). As a result, in orbital decompression surgery which removes the orbital wall in a rectangular shape, the stress at the orbital wall decreased as the width and depth of the removed orbital wall increased. In addition, in the case of orbital decompression, it can be seen that the chamfered model compared to the non-chamfered model (a form of general orbital decompression) have the stress reduction rate from 11.08% to 97.88%. It is inferred that if orbital decompression surgery considering the chamfered model is performed on an actual thyroid eye disease patient, it is expected that the damage to the extraocular muscle caused by the removed orbital wall will be reduced.

## 1 Introduction

Thyroid eye disease (TED) is an autoimmune disease characterized by lymphocyte infiltration in the orbit, including the extraocular muscle (EOM) and fat. In TED, the thyrotropin receptor antibodies (TRAbs) activate the immunological cascade in combination with the thyroid stimulating hormone receptor (TSH-R) on orbital fibroblasts, and they result in infiltration of activated B and T lymphocytes as well as fibrocytes that develop into myofibroblasts or adipocytes. The hydrophilic hyaluronic acid accumulation in the connective tissue and EOM can cause edema. Furthermore, the activation of periocular fibroblasts, which are known to be progenitor fat cells, can lead to the enlargement of orbital fat tissue (Gontarz-Nowak et al., 2020).

The TED has a worldwide prevalence rate of .1%–.3%, and has been reported to occur in approximately 40% of patients with Graves’ disease (Noth et al., 2001; Bartalena and Tanda, 2009; Mohyi and Smith, 2018). As the orbital inflammation progresses, swelling of the EOM and fat, increasing intraorbital pressure (IOP), proptosis, compressive optic neuropathy, and visual loss may occur (Fichter et al., 2012; Jefferis et al., 2018). Several treatment strategies are available, including highdose glucocorticoid therapy, orbital radiation therapy, and teprotumumab treatment, which is a novel insulin-like growth factor-1 receptor (IGF-1R) antibody (Bartalena et al., 1991; Mohyi and Smith, 2018). Surgical treatments are a multistage approach, and 20%–30% of TED patients are offered this option after the disease stabilizes. Orbital decompression is the first stage in sequential surgical treatment and is performed in inactive TED patients with severe proptosis, exposure keratopathy, facial disfigurement, persistent prolonged congestion, and high IOP (Ismailova et al., 2018). When vision is threatened by compressive optic neuropathy, emergent orbital decompression may be needed, even in the active TED phase (Wang et al., 2019). Despite these treatments, there is a possibility of disease recurrence owing to various reasons such as thyroid hormone instability, continuous smoking (Genere and Stan, 2019), and in some cases, orbital surgery itself, which can reactivate inflammation, exacerbate ophthalmopathy, postoperative motility disturbances, and cause EOM regrowth (Wenz et al., 1994; Alsuhaibani et al., 2011; Fichter et al., 2012; Wang et al., 2019).

Hu et al. (2010) observed a significant increase in medial rectus (MR) muscle volume and inferior rectus (IR) muscle mean volume postoperatively in TED patients who underwent orbital decompression surgery. Some hypotheses were suggested to explain the observed outcomes, such as the mild inflammatory reaction or the hydrostatic pressure changes due to the expanded orbital volume during surgery. However, the etiologies of these postoperative volumetric changes, motility disturbances, and disease reactivation are unclear (Wenz et al., 1994). Animal experiments or finite element analysis (FEA) can be used to identify and solve these problems indirectly. In particular, *in silico* methods using FEA have been extensively used for years to investigate disease mechanisms in other organs and in eye-related research. Baillargeon et al. (2014) mimicked a computer biomechanism-based cardiac model to extract pressure-volume relationships and conducted comparative studies with clinical observations. Schutte et al. (2016) simulated the rotation of the eyeball and EOM displacement due to torsion using the FEA technique and then analyzed the intraorbital eye behavior. Norman et al. (2011) proved the correlation between the sclera on the optic nerve head side and glaucoma. In addition, Fisher et al. (2021) conducted a gaze-evoked deformation study of optic nerve head deformities in TED patients. Although these studies on the eyeball and optic nerve behavior have been conducted using FEA, research on surgical orbital decompression has been limited. In addition, absolute standards for the location and method of orbital wall removal and the size or shape of the bone to be removed have not been quantitatively established in clinical studies. Thus, surgeries are based on the practicing clinicians’ experience.

Therefore, in this study, the behavior of EOM hypertrophy, which is one of the key contributors to elevated IOP, was simulated using the finite element method. An FEA technique was proposed to analyze the removed orbital wall’s location, size, and shape. Particular, this study focused on the IR muscle, which is one of the most common muscles affected in TED (Yoo et al., 2009), and examined the stress value of the orbital wall according to the hypertrophy of this muscle. TED can be classically divided into three types: type I (lipogenic), type II (myogenic), and type III (lipogenic + myogenic) (Hiromatsu et al., 2000). Among these types, type II is characterized by a hypertrophy of the EOM, which may result in restrictive myopathy. According to studies on the differential involvement of orbital fat and EOM, there were high cases of increased muscle volume while having a normal fat volume in patients with TED-related diseases (Forbes et al., 1986; Wiersinga et al., 2013). Therefore, this study hypothesized that the recurring EOM hypertrophy after surgery could cause reinflammation between the orbital wall and EOM. A series of computational simulations for pre- and post-operative EOM hypertrophy were performed to examine the stress variation from EOM hypertrophy. Theses stress results are overestimated to consider the safety in clinical applications. Furthermore, the location of the removed orbital wall was identified considering the maximum stress value, and the effects of the type and dimension of the removed orbital wall were examined.

## 2 Materials and methods

### 2.1 Analysis of major orbital dimensions based on magnetic resonance imaging

This study considered only TED patients having EOM variations. The major dimensions were measured based on the patient’s right orbit to examine the EOM variations in the TED-affected left orbit. The finite element model used in the series simulation was derived from medical images of a female patient in her 70s with moderate-to-severe, active TED at the initial diagnosis. The patient was treated with intravenous methylprednisolone, recommended by Bartalena et al. (2016). The patient was controlled for thyroid dysfunction in the Department of Endocrinology. The magnetic resonance image was acquired when TED was inactive, and no further progression of the disease for a year was confirmed. T1-weighted spin-echo images with a field of view (FOV) = 180 mm × 220 mm, matrix = 320 × 203, and slice thickness = 2.0 mm were acquired for magnetic resonance imaging (MRI). In addition, FOV/matrix was employed to establish pixel size. Coronal pictures were taken in 1 mm consecutive segments covering the entire orbit for the volume analysis. MRI were performed with the patient supine, and a stable head posture was achieved with adjustable head support. The patient underwent an MRI scan for 25–30 min with eyes closed and in a comfortable position. In addition, the numerical values of the intraorbital tissue were measured on the coronal, sagittal, and axial planes of the image. The study was conducted in accordance with the Declaration of Helsinki, and approved by the Institutional Review Board of Pusan National University Hospital (IRB No. 2104-018-102). All the patients voluntarily signed the informed written consent. Written consent to publish data containing personally identifiable information was also obtained.

**FIGURE 1:**
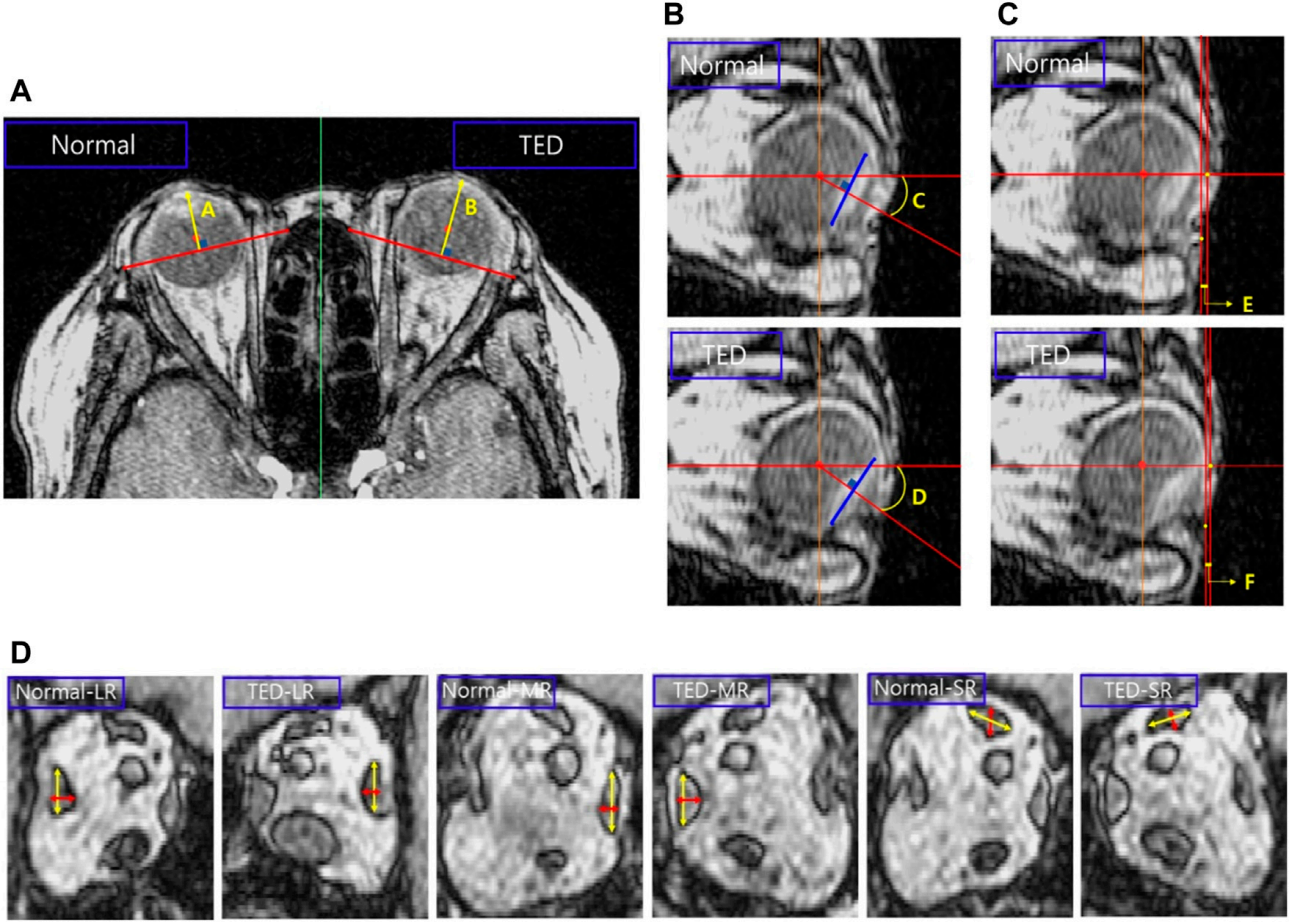
Analysis of major dimensions in the thyroid eye disease (TED) orbit and normal orbit based on magnetic resonance imaging (MRI). **(A)** Proptosis measurement. **(B)** Eye rotation measurement. **(C)** Distance measurement between the ocular and skin end point. **(D)** Thickness and width measurements in the extraocular muscle (EOM) except for the inferior rectus (IR) muscle.

As shown in Figure 1A, a straight line is drawn in the cross-sectional MRI image of the patient by connecting the end points of the zygomatic and maxillary bones. Another straight line is drawn perpendicular to this straight line, passing through the central point of the eyeball to measure proptosis. Comparing the proptosis dimensions of the two orbits confirmed that the proptosis in the TED orbit is 2.32 mm. Straight lines are drawn in the four azimuthal directions based on the center point of the eyeball in the sagittal plane of the patient’s MRI image, and a blue line is formed by connecting both ends of the eyeball lens, as in Figure 1B. After drawing a straight line in which the generated blue line is perpendicular to the eyeball center, the angle is measured. A comparison of the eye rotation dimensions of both orbits confirmed 6.84° eyeball rotation in the TED-affected orbit. Figure 1C shows straight lines perpendicular to the endpoint of the eyeball and lower skin in the patient’s sagittal plane. The measured distance between the two straight lines confirmed that the skin protruded by .29 mm in the TED-affected orbit. In addition, the distance in the TED orbit decreased by more than 27.36% compared to that in the normal orbit. Moreover, in Figures 1B, C, the measurements are in the sagittal plane corresponding to the lateral side of the zygomatic arch. Figure 1D shows the thickness and width measurements of the midpoints of each EOM in the coronal plane of the patient. In the TED orbit, the thickness and width loss rates are 7.58% and 5.58% in the MR muscle, −3.61% and .86% in the lateral rectus muscle, and −1.59% and 1.25% in the superior rectus muscle, respectively, compared with the normal orbit. Overall, the thickness of all EOMs increased, and the width decreased in the TED orbit compared with the normal orbit. In addition, all measurements in Figure 1 were repeated five times. The average values derived are listed in Table 1. These dimensions were used to select the load condition in the computerized IR hypertrophy and elevated IOP analyses.

**TABLE 1:** Dimensions measured in Figure 1.

Variables	A	B	C	D	E	F
Dimension	14.91 mm	17.23 mm	25.97°	32.81°	1.06 mm	.77 mm
	**LR**		**MR**		**SR**	
	**Normal**	**TED**	**Normal**	**TED**	**Normal**	**TED**
Thickness (mm)	3.60	3.73	3.43	3.69	3.78	3.84
Width (mm)	8.18	8.11	9.50	8.97	7.97	7.87

### 2.2 Modeling and material properties

**FIGURE 2:**
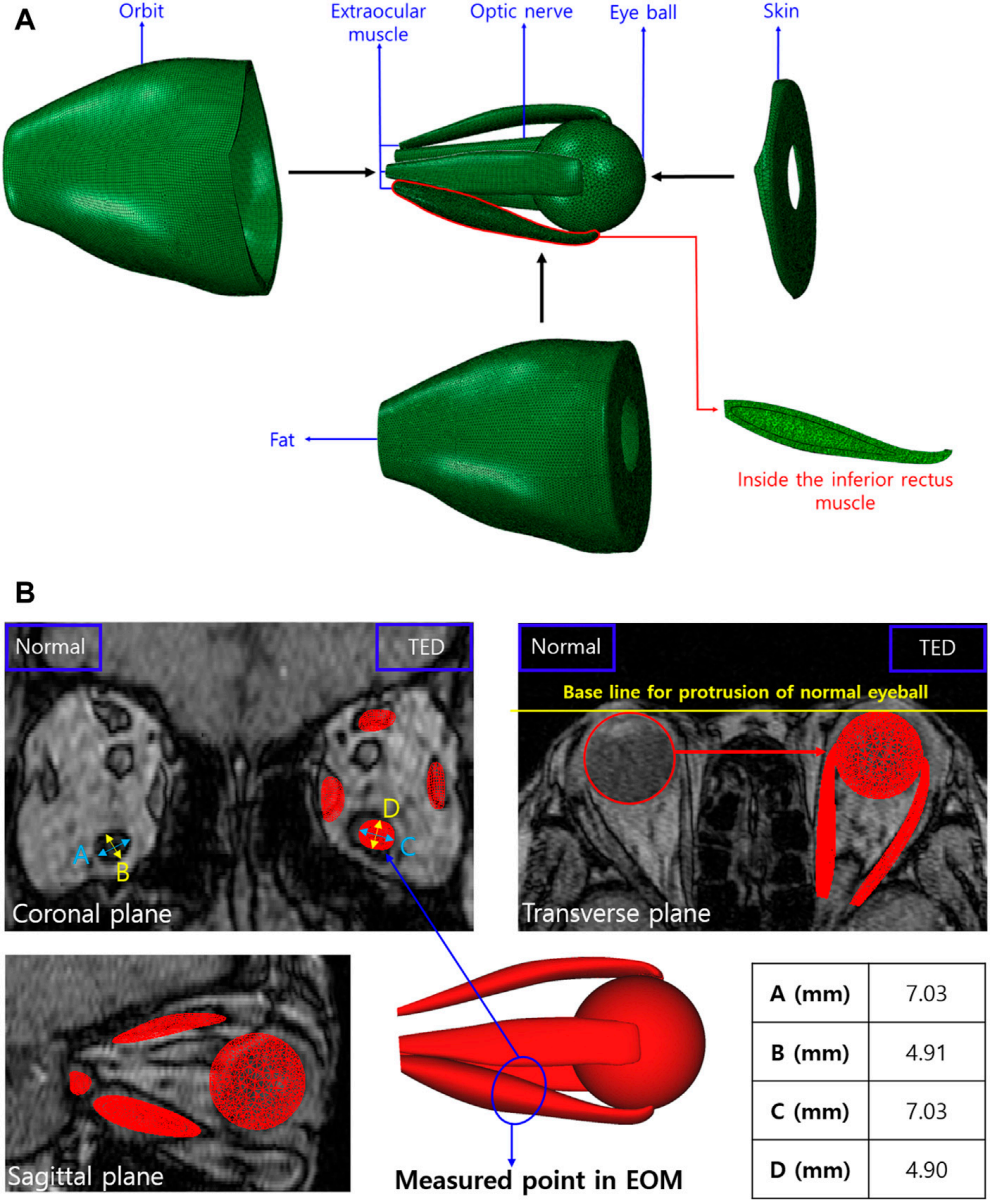
**(A)** Finite element model of orbit and intraorbital tissue. **(B)** Validation between the medical image and finite element model.

This study modeled the eyeball, orbit, EOM, optic nerve, skin, and fat as a finite element model using Mimics (version 19.0, Materialize, Leuven, Belgium), as shown in Figure 2A. The finite element model simulation, based on the patient’s medical image and the method proposed by Yiyi et al. (2005), offered a more precise simulation than the existing modeling method using MRI and computed tomography (CT) images. As shown in Figure 2B, the length and angle of the orbital medial wall (axial, coronal, and sagittal planes) and hypertrophied muscle dimensions obtained using the finite element model and medical image is compared to verify the model reliability. In the case of EOM, four rectus muscles are expressed, but in the case of the two oblique muscles, there is a model limitation in the expression, and they are excluded. In addition, a model resembling the IR shape is placed inside the IR, as shown in Figure 2A, for simulating the enlarged IR. When a load condition is applied only to the inner or outer parts of the IR, the actual hypertrophied IR shape could not be derived; thus, load conditions were applied to both parts simultaneously for analysis. Finally, ABAQUS (version 6.14, Dassault Systèmes, SIMULIA, United States) was used to perform three-dimensional FEA, and the finite element model consisted of 901,542 solid elements (C3D8R).

Table 2 lists the material properties for the elastic modulus (Ε), Poisson’s ratio (ν), and density (*ρ*) of each tissue applied in the FEA of TED patients. The fat surrounding the eye was assumed as nearly incompressible “soft” human tissue with ν = .49 (Power et al., 2002). In the literature on EOM, information on human material properties is lacking, and the material property values were estimated based on the tensile test results of the bovine EOM (Power et al., 2002; Schutte et al., 2006). The material properties that changed owing to IR hypertrophy were also based on literature (Barin et al., 2019). In FEA, the eyeball was set as a rigid body because only the proptosis and eye rotation measurements were significant, with no stress requirement. In addition, the material properties of the orbital wall, optic nerve, and skin investigated are listed in Table 2, based on existing studies (Cirovic et al., 2006; Geng et al., 2018).

**TABLE 2:** Material properties of finite element models.

Variables	Fat	EOMs	Optic nerve	Orbital wall	Eye ball	Skin
Ε (MPa)	.047	.09	5.5	14,500	14,500	1
ν	.49	0.4	.47	.35	0.3	.45
*ρ* (kg/m^3^)	999	1,600	1,012	1,610	—	—

### 2.3 Boundary and loading conditions

**FIGURE 3:**
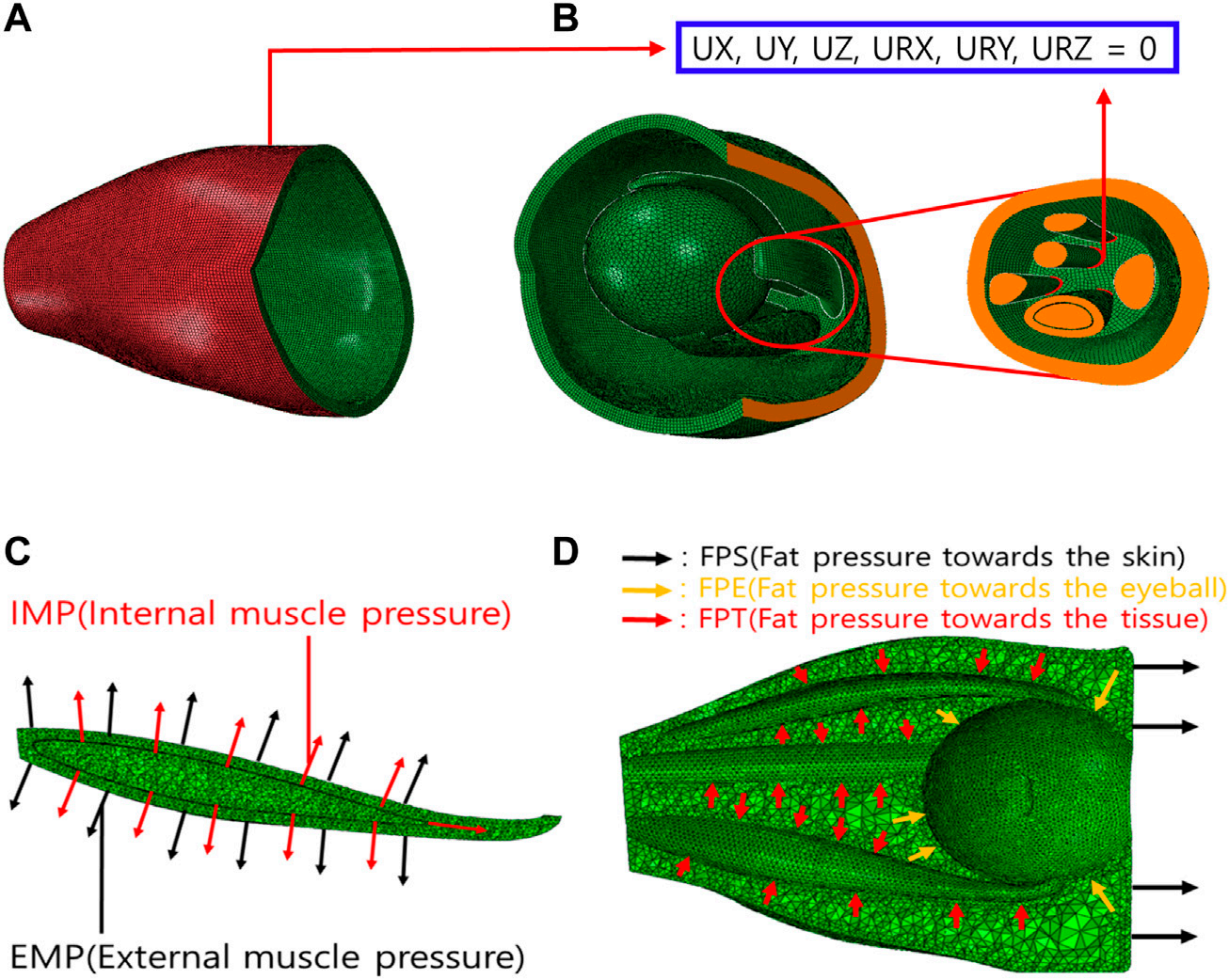
Boundary and load conditions of finite element model. **(A)** Orbital outer wall boundary conditions for movement restriction. **(B)** Boundary conditions of intraorbital. **(C)** Loading conditions of IR hypertrophy. **(D)** Loading conditions of elevated intraorbital pressure (IOP).

Figure 3 shows the boundary and load conditions of the finite element model. The orbital movement is restricted by securing the orbital outer wall (Figure 3A), and the degrees of freedom from the axial direction and rotation are restricted to the surface behind the orbital inner wall and that where the ocular and optic nerves begin (Figure 3B). The load condition is applied at two positions perpendicular to the IR surface (Figure 3C). Hypertrophy is simulated by generating internal and external muscle pressure (IMP and EMP) on the internal and external IR surfaces, respectively. In addition, fat pressure toward the skin (FPS), eyeball (FPE), and tissue (FPT) are generated to simulate IOP (Figure 3D). On average, the IOP of an adult is 3–6 mmHg (Kratky et al., 1990). In this study, the maximum value was set at 6 mmHg (equivalent to 800 Pa).

**FIGURE 4:**
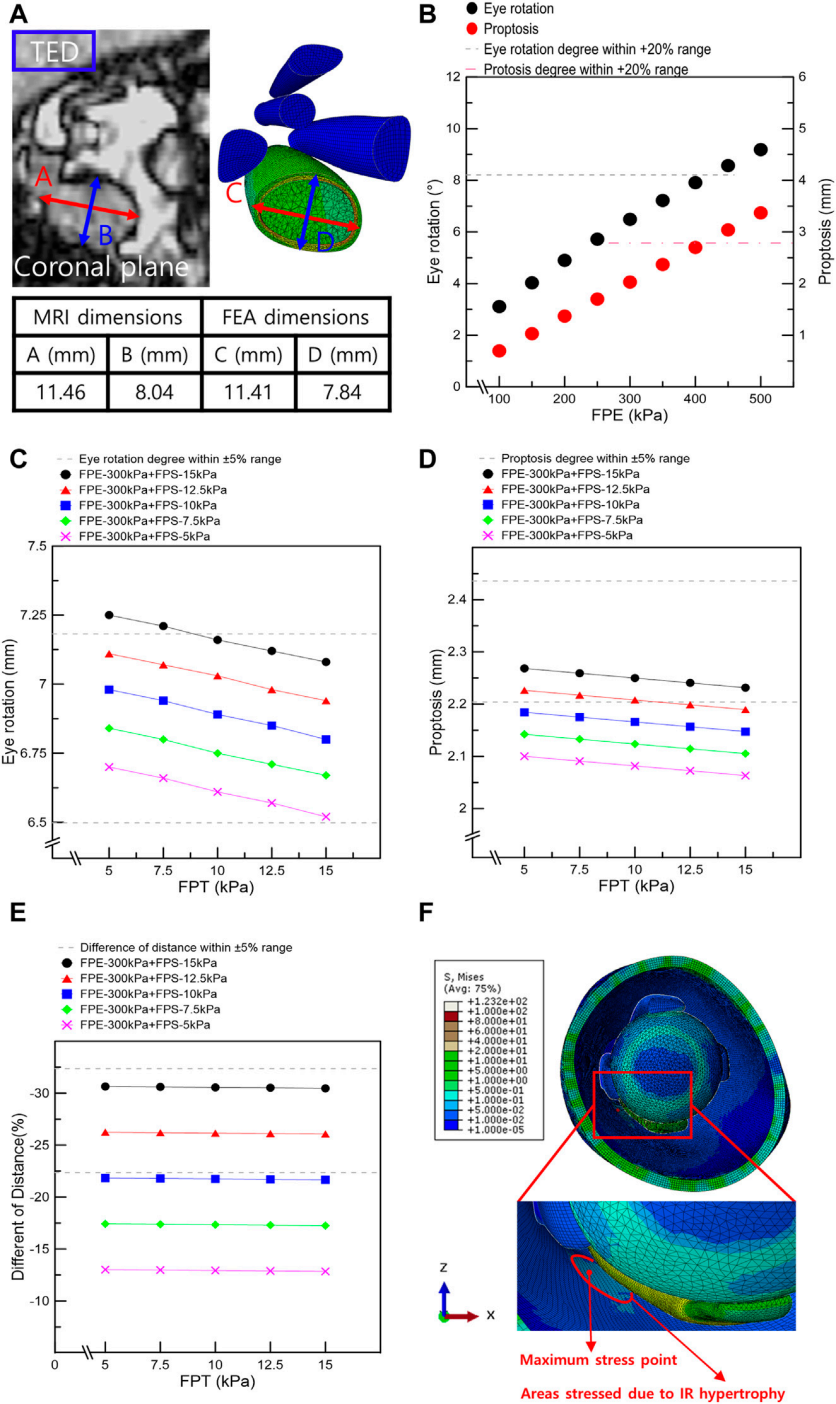
Results from finite element analysis of IR muscle hypertrophy and elevated IOP. **(A)** Thickness and width of IR in MRI image and finite element analysis of IR muscle hypertrophy. **(B)** Eye rotation and proptosis according to the load conditions of FPE. **(C)** Eye rotation responses according to the load conditions of fat pressure toward the skin eyeball (FPE), skin (FPS), and tissue (FPT). **(D)** Proptosis according to the load conditions of FPE, FPS, and FPT. **(E)** Distances between the ocular and skin endpoints according to the load conditions of FPE, FPS, and FPT. **(F)** Maximum stress point in FEA of IR hypertrophy and elevated IOP.

In addition, simulations were conducted under various loading conditions to simulate IR muscle hypertrophy and elevated IOP in TED patient’s. The results were validated based on the patient’s EOM hypertrophy, eye rotation, and proptosis values, as shown in Figure 4. First, IR hypertrophy was simulated by applying 45 and 300 MPa loads to the IMP and EMP. In FPE, which is most influenced by elevated IOP, simulations were conducted under a single loading condition, increasing from 100 to 500 kPa (interval: 50 kPa). The results under the loading condition of 100–400 kPa (interval: 50 kPa) were validated within the error range, set to +20% of the eye rotation and proptosis values measured by MRI. However, for more accurate simulation results, other complex loads such as FPS and FPT were considered. Second, 35 series simulations were conducted under FPE and FPS loading conditions ranging from 100 to 400 kPa (interval: 50 kPa) and 5–15 kPa (interval: 2.5 kPa), respectively. It was determined that the simulation results for the simultaneous FPE 300 kPa and FPS 5–15 kPa loading conditions were within the ±20% error range. Finally, 25 series simulations with loading conditions ranging from 5 to 15 kPa (interval: 2.5 kPa) of FPT to intraorbital tissues were performed, and cases within the ±5% error range were obtained.

### 2.4 FEA scenarios of orbital wall removal

The maximum stress point was identified at the optimal load conditions for the IR muscle hypertrophy and elevated IOP. Moreover, the orbital wall was removed (in rectangular or circular shapes), and a series FEA was performed. The results are given in Table 3. When the removed orbital wall is rectangular, a distance of 5 mm from the posterior surface of the orbital wall is assumed to be a risk area associated with the removal. Therefore, the posterior edge of the inner orbital wall is fixed at 8 mm to facilitate the removal of the rectangular orbital wall. Subsequently, the length of the orbital wall front edge and the depth of the rectangle to be removed were changed, and FEA was performed according to the presence or absence of chamfering. When the removed orbital wall was circular, the maximal stress point in the IR muscle hypertrophy and elevated IOP FEA was set as the circle’s center point. Subsequently, FEA was performed according to the circle diameter and the presence or absence of a chamfer.

**TABLE 3:** Scenarios associated with orbital wall removal.

	Rectangular		
No.	Chamfering	Width (mm)	Depth (mm)
1	X	10	15–25 (Interval: 2)
2	X	12	15–25 (Interval: 2)
3	X	14	15–25 (Interval: 2)
4	O	10	15–25 (Interval: 2)
5	O	12	15–25 (Interval: 2)
6	O	14	15–25 (Interval: 2)
	**Circular**		
	**Chamfering**		**Diameter (mm)**
7	X		10–15 (Interval: 1)
8	O		10–15 (Interval: 1)

In addition, CT scans of four TED patients who had orbital decompression were evaluated to identify the volumetric changes and possible damage to EOM to assess the TED recurrence risk. The four male patients (aged 46–65) had their orbital floor and medial wall removed. The inclusion criteria were evidence of disease inactivity for at least 1 year, normal thyroid function, and normal thyroid-stimulating hormone receptor antibodies, including thyroid-stimulating antibodies. Modified NOSPECS classification scores determined clinical severity, and the Clinical Activity Score (CAS) was used to assess the TED activity (Bartalena et al., 2016).

## 3 Results

### 3.1 Selection of orbit loading conditions based on IR muscle hypertrophy

Simulations were performed based on the above-mentioned scenarios to simulate the IR hypertrophy and elevated IOP in TED patients. Figure 4A shows the outcomes of the IR muscle hypertrophy as measured by the FEA and the IR muscle dimensions of the TED patient as measured using the CT. In addition, the aforementioned loading conditions were used to simulate IOP and IR muscle hypertrophy, and Figures 4B–E display the results as eye rotation, proptosis, and skin protrusion. Especially, Figure 4B shows the procedure for obtaining outcomes within a +20% error range during the initial selection phase for FPE loading conditions. Figures 4C–E also demonstrate the established results based on the medical information of TED patients and computational simulations; 69 scenarios were simulated with FPE, FPS, and FPT loading conditions. In Tables 4, 5, six scenarios had an error rate of less than 5% compared to the MRI-measured dimensions. In these six scenarios, the same ranges of concentrated stresses were obtained from the inferomedial orbital strut to the rear one-third point (Figure 4F). In addition, in the six scenarios, the maximum stress in the region of concentrated stress was measured at the same point.

**TABLE 4:** Muscle loss rate in finite element analysis of inferior rectus (IR) muscle hypertrophy and the elevated IOP (based on FPE of 300 kPa).

			FPS-12.5 kPa			FPS-15 kPa		
		Base dimension before analysis	FPT 7.5 kPa	FPT 10 kPa	FPT 12.5 kPa	FPT 10 kPa	FPT 12.5 kPa	FPT 15 kPa
Thickness (mm)	LR	3.80	3.87	3.85	3.83	3.85	3.83	3.81
	MR	4.12	4.47	4.44	4.41	4.44	4.42	4.39
	SR	3.47	3.60	3.58	3.56	3.58	3.56	3.54
Width (mm)	LR	7.71	7.39	7.36	7.32	7.36	7.33	7.29
	MR	7.71	7.47	7.43	7.39	7.43	7.40	7.36
	SR	7.15	7.14	7.11	7.08	7.11	7.08	7.05
	**Loss rate measured using MRI**							
Thickness (%)	LR	−3.61	−1.79	−1.29	−.78	−1.37	−.87	−.37
	MR	−7.58	−8.39	−7.68	−6.97	−7.87	−7.16	−6.45
	SR	−1.59	−4.01	−3.38	−2.74	−3.43	−2.79	−2.16
Width (%)	LR	.86	4.09	4.57	5.04	4.47	4.95	5.42
	MR	5.58	3.14	3.64	4.14	3.54	4.04	4.55
	SR	1.25	.21	.65	1.09	.56	1.00	1.45

**TABLE 5:** Main dimensions of IR hypertrophy and the elevated IOP simulation.

Case no.	FPE (kPa)	FPS (kPa)	FPT (kPa)	Distance difference between the eye and skin endpoint		IR thickness (mm)	IR width (mm)	Eye rotation (°)	Proptosis (mm)	Orbital wall stress (kPa)
				(mm)	(%)					
1	300	12.5	7.5	1.0319	−26.19	7.84	11.41	7.07 (Down)	2.22 (Protend)	117.44
2	300	12.5	10	1.0324	−26.15	7.84	11.41	7.03 (Down)	2.21 (Protend)	117.47
3	300	12.5	12.5	1.0330	−26.11	7.84	11.41	6.98 (Down)	2.20 (Protend)	117.50
4	300	15	10	.9709	−30.55	7.84	11.41	7.16 (Down)	2.25 (Protend)	117.09
5	300	15	12.5	.9714	−30.52	7.84	11.41	7.12 (Down)	2.24 (Protend)	117.12
6	300	15	15	.9721	−30.46	7.84	11.41	7.08 (Down)	2.23 (Protend)	117.15

### 3.2 Stress distribution and trends in rectangular orbital wall removal

**FIGURE 5:**
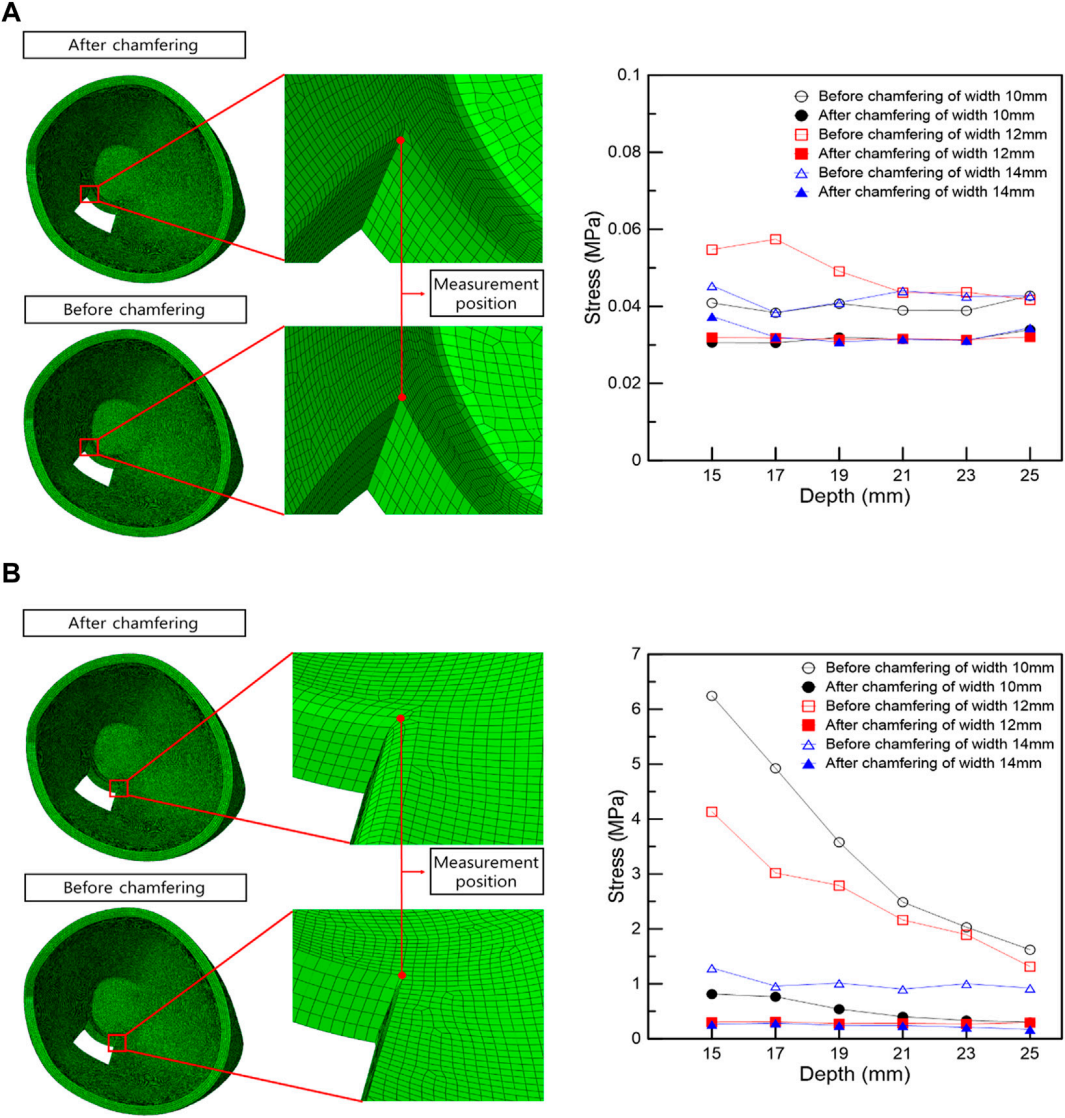
Stress measurement position and stress graph in the vertex following the rectangular removal of the orbital wall. **(A)** Left vertex. **(B)** Right vertex.

The optimal loading conditions were identified in the FEA of IR hypertrophy and elevated IOP in TED patients. Subject to the optimal load conditions, the range was set in a rectangular shape based on the maximum stress point in the orbit wall, the orbital wall was removed, and the surrounding stress was measured. Stress was measured at the left and right vertices of the inner corner of the orbit, which are common in rectangular orbital wall removal scenarios. Figure 5A shows that the stress before and after chamfering at the left vertex has no significant effect, depending on the removal depth and width. However, the FEA results before and after chamfering at the right vertex indicate a slight difference in stress.

In the right vertex, in Figure 5B, the stress decreased as the width and depth of the removed orbital wall increased, and the stress difference before and after chamfering was identified. In addition, the chamfered model analysis confirmed low-stress levels, regardless of the depth and width, in most cases. Therefore, in the right vertex, a large stress difference exists depending on chamfering rather than the depth or width removed, as shown in Table 6.

**TABLE 6:** Induced stress at left and right measurement positions following the removal of the orbital wall with a rectangular shape.

Depth (mm)	Width (mm)	Stress of left point (MPa)		Stress of right point (MPa)	
		Before chamfering	After chamfering	Before chamfering	After chamfering
15	10	.035326	.03062	6.24453	.813527
	12	.054737	.032	4.13248	.302808
	14	.04535	.03740	1.28819	.259452
17	10	.038368	.03053	4.92509	.764399
	12	.057419	.03179	3.02016	.307206
	14	.038398	.03199	.95818	.284504
19	10	.040739	.03198	3.57721	.538666
	12	.04911	.03143	2.79114	.276484
	14	.041005	.0308	1.01248	.241615
21	10	.038983	.03153	2.48868	.400518
	12	.043538	.0316	2.16375	.282113
	14	.044043	.03151	.90297	.238358
23	10	.038903	.03126	2.03128	.33216
	12	.035255	.03135	1.89548	.26846
	14	.090272	.03116	1.011	.20817
25	10	.042763	.03394	1.621	.300002
	12	.041728	.0321	1.31453	.2928
	14	.042743	.03444	.92155	.169208

### 3.3 Stress distribution and trends following the circular orbital wall with removal

**FIGURE 6:**
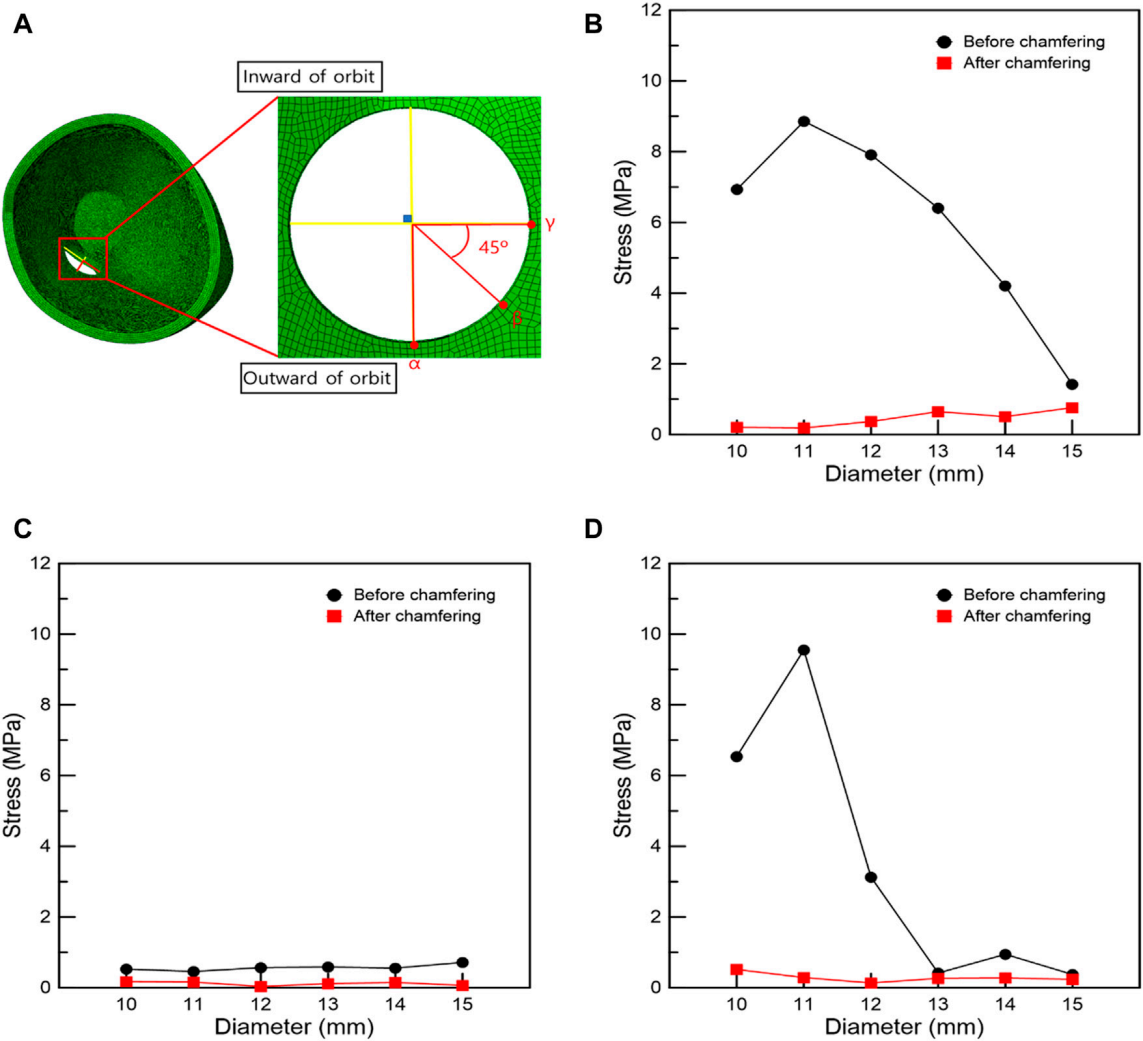
Stress measurement position and graphs associated with the circular removal of the orbital wall. **(A)** Stress measurement position. Stresses at positions **(B)** *γ*, **(C)** *α*, and **(D)** *β*.

The maximum stress point in the FEA of IR hypertrophy and elevated IOP was at the circle center in circular orbital wall removal. Moreover, a circular orbital wall was removed owing to the increase in diameter before and after chamfering, and the surrounding stress was measured. The stress measurement position was divided along four directions, as shown in Figure 6A, and stress measurement was performed at three points by selecting the direction with a high-stress distribution rate. FEA of the orbital wall removal model before chamfering, as shown in Figures 6B–D, confirmed that point *α* had lower stress than the other points owing to the distance from the maximum hypertrophy point of the IR. In addition, at *β* and *γ* points, the stress decreases as the diameter increases. Accordingly, the high stress is measured when the removed orbital wall diameter is 11 mm.

**FIGURE 7:**
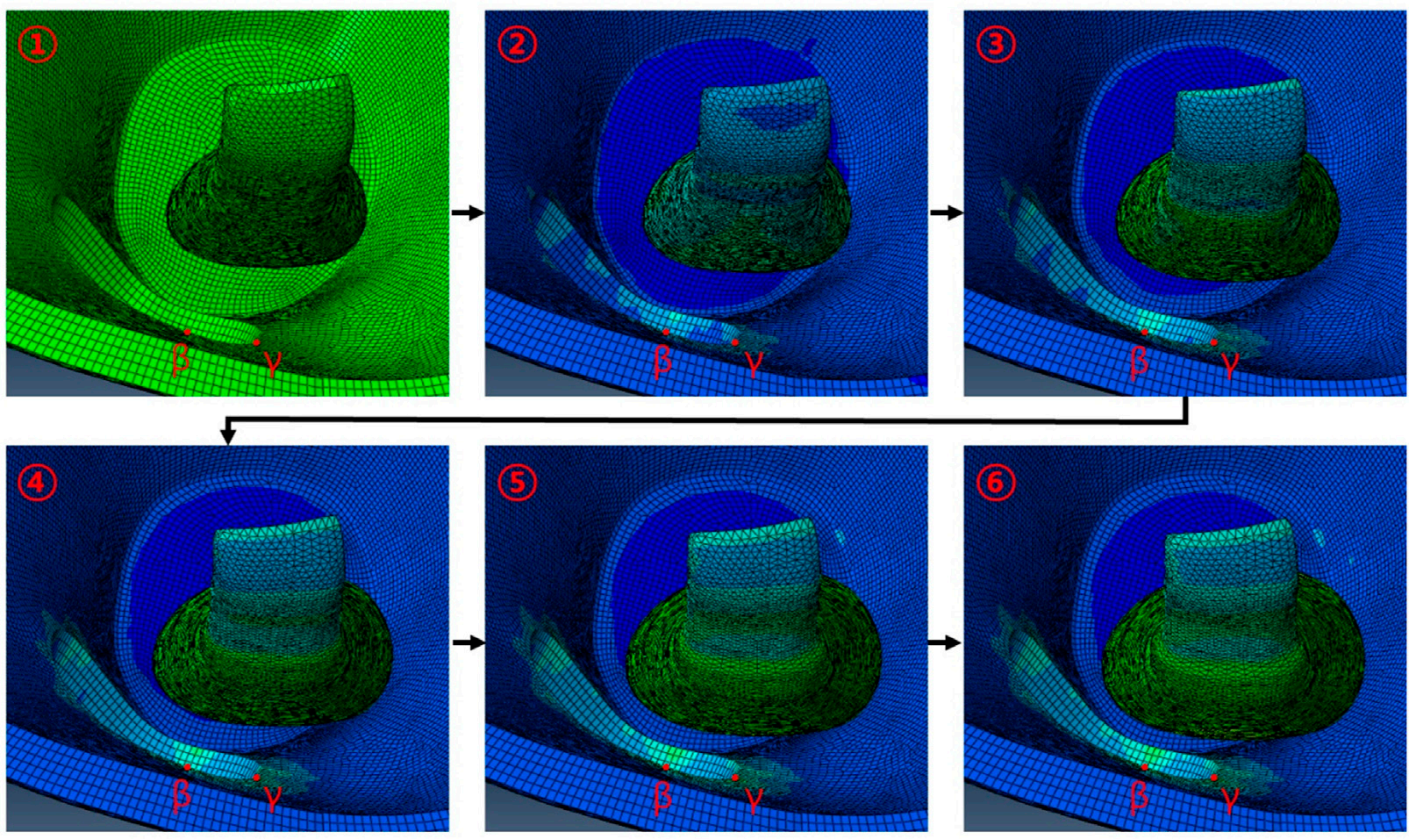
Behavior associated with IR hypertrophy in FEA following circular orbital wall removal of diameter 11 mm.

As shown in Figure 7, when IR is hypertrophic, the positions of the *β* and *γ* points coincide with the point of maximum hypertrophy when the removed orbital wall is 11 mm in diameter, and the highest stress load is received. Compared to the model with 11 mm diameter, the load applied to the *β* and *γ* points was reduced as the diameter decreased or increased in models with diameters in the range of 10–15 mm. Therefore, it was confirmed that the IR stress was highest near the maximum hypertrophy point of the IR at *β* and *γ* points. In addition, in the chamfered orbital wall removal model analysis, the stress was lower than that before chamfering at *α*, *β*, and *γ* points, as shown in Table 7.

**TABLE 7:** Stress at *α*, *β*, and *γ* positions when removing the orbital wall in a circular shape.

Diameter (mm)	Stress at *α* point (MPa)		Stress at *β* point (MPa)		Stress at *γ* point (MPa)	
	Before chamfering	After chamfering	Before chamfering	After chamfering	Before chamfering	After chamfering
10	.528024	.1746	6.536398	.516669	6.935254	.207131
11	.459065	.159243	9.551042	.287233	8.859715	.187805
12	.567837	.035651	3.12807	.134126	7.912384	.370978
13	.58731	.114341	.41591	.2646	6.40473	.649128
14	.554781	.150457	.945533	.277382	4.206018	.507885
15	.71653	.064393	.37459	.23486	1.42209	.761282

### 3.4 Computed tomographic analysis of patient datasets with removed orbital wall

**FIGURE 8:**
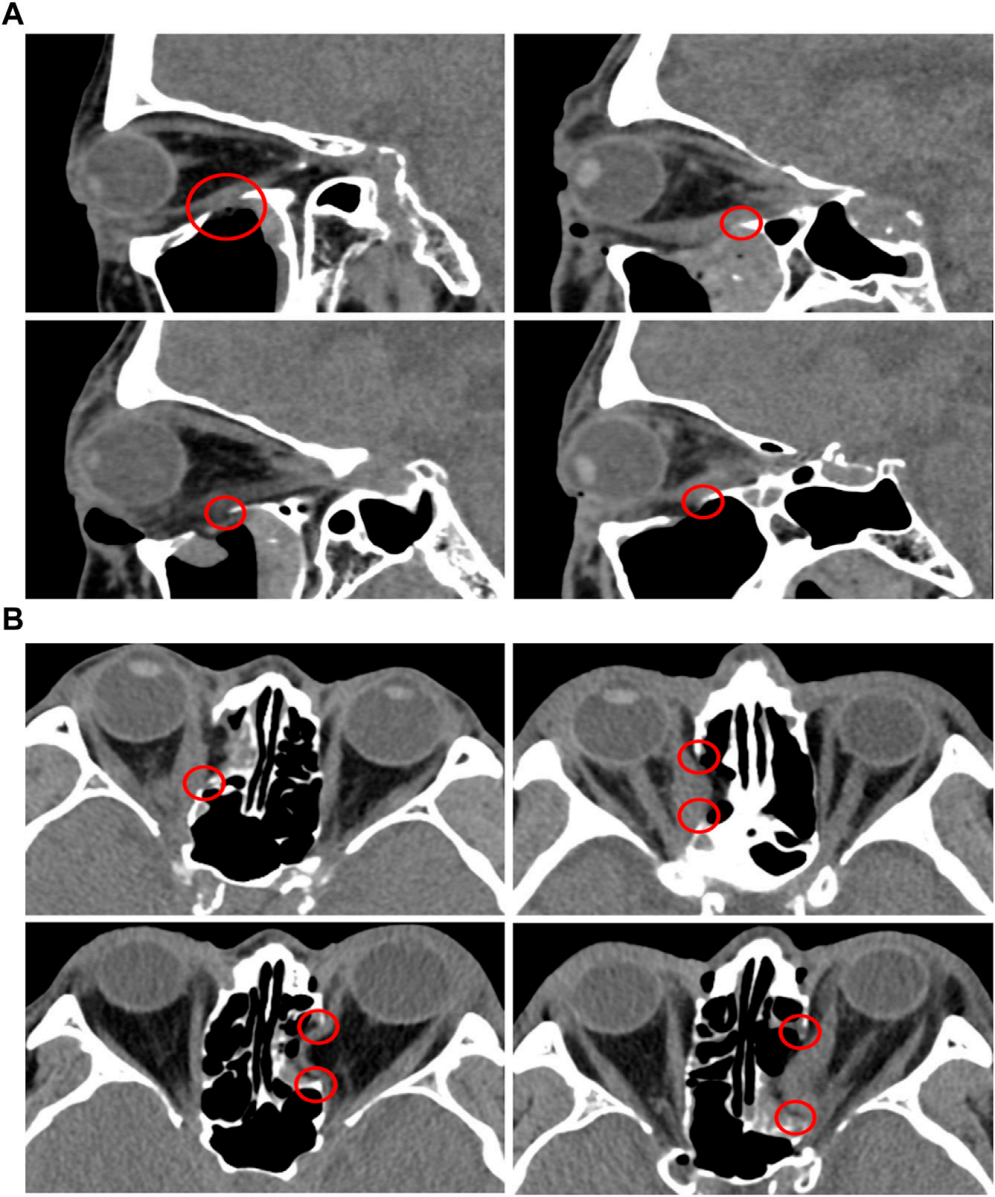
CT analysis of TED patients who underwent orbital decompression surgeries. **(A)** Sagittal plane. **(B)** Axial plane.

The risk of damaging EOMs and recurrence of TED at the surgery site was evaluated using image analysis, as shown in Figure 8. Based on sagittal and axial CT images, Figure 8 shows the orbital tissues of the four patients whose orbital floor and the medial wall had been previously removed. As depicted in Figure 8, the EOM and surgical site’s margin with a sharply sloping surface were observed. In other words, the space between the hypertrophied EOM and the margin of the surgical site is extremely narrow. Particularly, as a large quantity of EOM moves toward the decompression site, the EOM surface at the surgical site edge becomes compressed and sharper. After orbital decompression, the EOM and bone margin became contiguous, and the EOM underwent considerable morphological alteration.

## 4 Discussion

TED is an autoimmune inflammatory disease characterized by proptosis attributed to EOM. Accordingly, orbital decompression is required to remove the orbital wall and lower IOP. We simulated the IR hypertrophy and elevated IOP of TED patients and identified the maximum stress point of the orbital wall. Subsequently, FEA was performed based on the maximum stress point of the removed orbital wall according to the shape and size, and stress changes were confirmed.

First, we analyzed the MRI images of TED patients and constructed a three-dimensional finite element model; the IR model before hypertrophy was implemented by analyzing the IR dimension in the patient’s normal orbit. In addition, a model similar to the IR shape was created inside IR to show the hypertrophic response of the IR muscle. When the load value was defined only inside or outside the IR muscle, the shape of the actual hypertrophic IR muscle could not be reproduced; thus, both loads were applied simultaneously. With only hypertrophic IR muscle, the eye rotation and proptosis could not be measured; therefore, the load condition was applied vertically to the surfaces associated with the intraorbital tissue, and IOP variations were simulated. The results confirmed that the thickness and width of the IR muscle, proptosis, eye rotation, distance from the skin and the eyeball endpoint, and the muscle’s loss rate were associated with an error rate of ±5% according to the dimension measured on MRI image. In addition, the maximum stress point on the orbital wall owing to IR hypertrophy and elevated IOP was determined to be the location where clinical surgery was performed. The results confirm that the FEA of IR hypertrophy and elevated IOP performed in this study are reliable.

In the FEA of the IR hypertrophy and elevated IOP, orbital decompression was simulated by the rectangular orbital wall removal based on the maximum stress point. A distance of 5 mm anterior from the posterior orbital inner wall surface was assumed as a risk area for orbital wall removal. This is because when removal was attempted at the back of the orbital wall, it increased the damage risk to critical structures at the orbital apex, and it is difficult to access to the end of the orbital wall clinically. In addition, the stress at the inner vertex of the non-chamfered model, and that in the middle of the edge connected to the inner vertex in the chamfered model were measured to evaluate the stress trend according to the removed depth and width. This was set as the stress measurement location because it is the only location that remains the same even if the depth and width of the orbital wall to be removed in the rectangular orbital decompression FEA changed. It was observed that the load decreased on the right vertex as the values of the removed depth and width increased, and large load stress was applied to the right vertex before chamfering compared with the post-chamfering state. However, in the left vertex, there was no significant difference in stress with depth and width, and similar stress was applied before and after chamfering. This is probably because the load transmission was weakened as the position of the hypertrophic IR was farther away.

Additionally, in the FEA of the IR hypertrophy and elevated IOP, orbital decompression was simulated in which the maximum stress point was set to the distal center, and the orbital medial wall was removed in a circular shape. Four orientations were set in the circle for circular orbital decompression, and three points were selected in the area with the highest generated stress to measure the stress. This is because measuring the stress at the same location as in the rectangular orbital decompression procedure with increasing diameter becomes challenging. As the distance from the hypertrophic IR increased, the load applied to the *α* position decreased, and a low-stress level was measured. The difference in stress before and after chamfering was insignificant. At *β* and *γ* points, the load decreased as the diameter increased. This was because it coincided with the point of maximal IR hypertrophy. Consequently, it was proved that when the removed circle diameter decreased or increased by more than 11 mm, it moved away from the point of maximum IR hypertrophy and resulted in low-load stress values. However, there was no decrease in the stress of the orbital wall with the increasing diameter after chamfering. These findings confirmed that the stress bands were similar. Hence, it was confirmed that the non-chamfered model was significantly affected by the hypertrophic IR muscle compared to the chamfered model, regardless of the removed orbital wall shape.

In addition, we analyzed the CT images of TED patients who underwent orbital decompression to understand TED recurrence risk. The removed margin of the orbital medial wall and margin of the orbital floor had a steep slope and were confirmed to be in close contact with the EOMs. Hu et al. (2010) reported that the volume of IR and MR increased after orbital decompression surgery. Therefore, it was concluded that inflammation was generated in IR and MR owing to the close contact between the bone edge and muscle, which is one of the causes of TED recurrence. There is a need for a method to minimize EOM damage to prevent TED recurrence, considering the significant level of damage caused.

In the non-chamfered orbit model, the shape of the bone margin removed during orbital decompression in actual surgery was similar. Large stress was confirmed in the non-chamfered orbital wall based on the simulation of the orbital decompression through FEA. Therefore, we reaffirmed the concern that sharp edges severely damage the EOM. The chamfered orbital decompression is proposed as the first method to minimize EOM damage. The proposed method significantly reduces the stress applied to the orbital wall compared with that in non-chamfered orbital decompression. Therefore, when implementing orbital decompression and chamfering by drilling, the stress applied to the EOM is reduced, and the damage is minimized. However, there are cases wherein it is difficult to implement chamfering because the orbital wall is thin. Hence, another method is required to protect the sharp edges of the margin.

Therefore, we provide a method of incising the periorbita between the orbital wall and fat in a specific form to protect the sharp edges. Periorbita exists between the orbital wall and fat; as the periorbita provides suspension for the orbital contents not to prolapse even after orbital wall removal, the periorbital incision is the key procedure during orbital decompression surgery. Some methods are reported on periorbita incisions like complete removal of the periorbita after removing the orbital wall (Cubuk et al., 2018; Jefferis et al., 2018), the orbital sling procedure method that preserves the median part of the periorbita to keep the MR muscle intact (Metson and Samaha, 2002), and the method of making a parallel incision of the periorbita from the posterior to the anterior (Jimenez-Chobillon and Lopez-Oliver, 2010; Hernandez-Garcia et al., 2017). The exposure of bare bone without periorbital covering through the complete removal of periorbita does not protect the edge of the removed orbital wall and can damage the EOM. The orbital sling procedure might have some protective function but diminishes the extent of decompression. We propose a method in which the periorbita is incised in the II-shape in parallel from the posterior to the anterior direction, and incisions are made in the direction perpendicular to the incised line to perform H-shaped incision. Therefore, we conclude that the “H” shaped periorbita will cover both sides of the sharp edge of the removed orbital wall and minimize the EOM damage.

However, this study had several limitations. First, the inferior oblique muscle and superior oblique tendon exist in addition to the four rectus muscles inside the orbit, and various biological tissues, such as ligaments, septum, and lacrimal gland, are complexly located inside the orbit. Biological tissues, such as oblique muscles and tissues, were excluded from this analysis because the location and exact dimensions could not be determined from the MRI and CT data. It is believed that more accurate results can be obtained by simulating the oblique muscle and tendon. However, behavior inside the orbit of a TED patient can be efficiently simulated with the four rectus EOMs. Second, the material properties of the finite element model of the orbit and intraorbital tissues were assumed based on animal experiments because of the lack of experimental literature and information on human properties. Therefore, the accuracy of the FEA results can be further improved by obtaining the human material properties. However, there are ethical issues associated with the conduct of *in vivo* experiments. Therefore, in this study, the physical properties of the orbit and intraorbital tissues were applied using the results of published animal experimental studies. Third, the eyeball may be deformed by IR hypertrophy, considering that it has various structures and its inside is filled with vitreous and aqueous humor. However, this study focused on examining the contact stress of the orbital inner wall, excluding the eyeball, caused by EOM hypertrophy. If the deformation of other tissues, such as the eye, is considered, further results can be achieved besides the contact stress in the inner orbital wall. Finally, if image data is available prior to the patient’s onset of thyroid eye disease, more accurate results for muscle growth variation can be obtained. However, because medical images were difficult to obtain prior to the onset of thyroid eye disease in this study, the main dimensions were measured based on the patient’s contralateral orbit.

## 5 Conclusion

We simulated the orbit and intraorbital tissue of TED patients with enlarged IR muscle with a finite element model. In addition, an FEA was performed to assess the tissue responses in the orbit of TED patients based on the application of load conditions to the IR muscle and IOP. Thereafter, FEA of the tissue behavior in the orbit of a TED patient was performed after the orbital wall removal around the point of maximum stress on the orbital lining under the same conditions. The orbital wall stress at a specific point was measured according to the shape and location of the removed orbital wall. The main conclusions of this study are as follows.

• The hypertrophic load conditions of the IR muscle alone could not simulate the rotation and extrusion of the eye; the addition of IOP load conditions could simulate the intraocular tissue behavior of TED patients.
• The maximum stress point of the orbital wall was identified by the FEA of the IR hypertrophy and elevated IOP, and this point was applied for surgery.
• When the removed orbital wall has a rectangular shape, the difference in stress before and after chamfering was small at locations farther than the IR hypertrophy position. However, non-chamfered model analysis at a point close to the IR hypertrophy position confirmed that the applied stress decreased as the depth and width of the removed orbital wall increased.
• In chamfered model analysis with rectangular orbital wall removal, the stress decreased as the overall removal depth and width increased, but the stress width was not large. In addition, the orbital wall stress of the non-chamfered model with general orbital decompression formed a higher load than the orbital wall stress of the chamfered model.
• In the non-chamfered model with circular orbital wall removal, the stress of the orbital wall decreased as the diameter increased. However, the highest stress was measured at a specific diameter close to the maximum IR hypertrophy point. In the chamfered model, there was no difference in stress with the diameter of the removed circle.
• When the removed orbital wall had a circular shape, the stress of the non-chamfered model, with general orbital decompression, was significantly greater than that of the chamfered model.
• Therefore, we confirmed that the edge of the removed orbital wall, regardless of the removal type, generated significantly higher stress on the orbital wall in the case of the non-chamfered model (a form of general orbital decompression) than that in the chamfered model.
• Finally, if the edge of the orbital wall removed during orbital decompression was removed in a chamfering form, the EOM damage was expected to be minimal, preventing recurrence. If the orbital wall was thin and could not be removed in a chamfering form, the periorbita between the orbital wall and fat could be sectioned in an “H” shape. Moreover, the orbital wall of the sharp edge would be wrapped with the periorbita to prevent EOM damage.

Thus, if orbital decompression surgery is performed on a TED patient, the damage to the EOM caused by the removed orbital wall could be reduced. In addition, even if TED recurs, EOM damage can be minimized. Overall, the findings and inferences of this study can help treat TED in patients. Furthermore, only the hypertrophied IR in TED patients was considered, but in future studies, complex environmental conditions, including hypertrophied cases of other EOMs, will be included.

## Data Availability

The original contributions presented in the study are included in the article/Supplementary Material, further inquiries can be directed to the corresponding authors.
